# Concentric Ring Trajectory Sampling With k‐Space Reordering Enables Assessment of Tissue‐Specific *T*_1_ and *T*_2_ Relaxation for ^2^H‐Labeled Substrates in the Human Brain at 7 T

**DOI:** 10.1002/nbm.5311

**Published:** 2024-12-19

**Authors:** Viola Bader, Bernhard Strasser, Wolfgang Bogner, Lukas Hingerl, Sabina Frese, Anna Duguid, Aaron Osburg, William T. Clarke, Stanislav Motyka, Martin Krssak, Siegfried Trattnig, Thomas Scherer, Rupert Lanzenberger, Fabian Niess

**Affiliations:** ^1^ High Field MR Center, Department of Biomedical Imaging and Image‐Guided Therapy Medical University of Vienna Vienna Austria; ^2^ Christian Doppler Laboratory for MR Imaging Biomarkers (BIOMAK), Department of Biomedical Imaging and Image‐Guided Therapy Medical University of Vienna Vienna Austria; ^3^ Wellcome Centre for Integrative Neuroimaging, FMRIB, Nuffield Department of Clinical Neurosciences University of Oxford UK; ^4^ Department of Medicine III, Division of Endocrinology and Metabolism Medical University of Vienna Vienna Austria; ^5^ Institute for Clinical Molecular MRI Karl Landsteiner Society St. Pölten Austria; ^6^ Department of Psychiatry and Psychotherapy, Comprehensive Center for Clinical Neurosciences and Mental Health (C3NMH) Medical University of Vienna Austria

**Keywords:** ^2^H MRSI, 3D magnetic resonance spectroscopic imaging, deuterium metabolic imaging, DMI, glucose metabolism, relaxation time measurements

## Abstract

Deuterium metabolic imaging (DMI) is an emerging Magnetic Resonance technique providing valuable insight into the dynamics of cellular glucose (Glc) metabolism of the human brain in vivo using deuterium‐labeled (^2^H) glucose as non‐invasive tracer. Reliable concentration estimation of ^2^H‐Glc and downstream synthesized neurotransmitters glutamate + glutamine (Glx) requires accurate knowledge of relaxation times, but so far tissue‐specific *T*_1_ and *T*_2_ relaxation times (e.g., in gray and white matter) have not been determined. Such measurements are time‐consuming and particularly challenging in the presence of dynamically changing metabolite levels (e.g. ^2^H Glc and ^2^H Glx).

This study aimed to assess *T*_1_ and *T*_2_ relaxation times of deuterated resonances, i.e., water, Glc and Glx in human gray and white matter using inversion recovery and Hahn spin‐echo ^2^H MRSI (magnetic resonance spectroscopic imaging), respectively, with non‐Cartesian concentric ring trajectory readout (CRT) including specific k‐space reordering at 7 T. The sequence was validated using phantom measurements and all results were compared to unlocalized acquisitions. Thirteen healthy volunteers participated in the study, with 10 of them scanned ~90 min after oral administration of 0.8 g/kg [6,6′‐^2^H]‐glucose. Significantly different *T*_1_ and *T*_2_ relaxation was observed between GM and WM for ^2^H water (*T*_1_^GM/WM/unlocalized^ = 358 ± 21/328 ± 12/335 m ± 6 ms*, p* = 0.01) and ^2^H Glx (*T*_2_^GM/WM/unlocalized^ = 37 ± 2/35 ± 2/33 ± 3 ms*, p* = 0.02), respectively, consistent with unlocalized acquisitions. No significant regional differences were found for ^2^H water (*T*_2_^GM/WM/unlocalized^ = 36 ± 2/34 ± 2/31 ± 2 ms, *p* = 0.08), ^2^H Glc (*T*_1_^GM/WM/unlocalized^ = 70 ± 5/73 ± 4/80 ± 5 ms, *p* = 0.13; *T*_2_^GM/WM/unlocalized^ = 36 ± 1/34 ± 2/34 ± 2 ms, *p* = 0.24) and Glx (*T*_1_^GM/WM/unlocalized^ = 172 ± 15/172 ± 12/165 ± 11 ms, *p* = 1.00).

Knowledge of tissue‐specific relaxation times can enhance the accuracy of concentration estimation and metabolic flux rates in future studies, potentially improving our understanding of various brain diseases such as cancer, neurodegenerative diseases or diabetes, which are often linked to impaired glucose metabolism.

## Graphical abstract

Inversion recovery and Hahn spin‐echo acquisition schemes were implemented into 3D FID ^2^H‐MRSI using fast concentric ring trajectory readout with k‐space reordering to measure tissue‐specific relaxation times of deuterated resonances in the human brain at 7 T (water, glucose, and glutamine + glutamate).

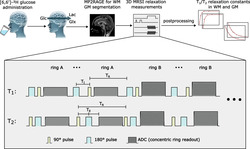

**Abbreviations**
ATP
: adenosine triphosphate
COV
: coefficient of variation
CRT
: concentric ring trajectory
CSI
: chemical shift imaging
DMI
: deuterium metabolic imaging
FID
: free induction decay
Glc
: glucose
Glx
: combined glutamine+glutamate
GM
: gray matter
Lac
: lactate
MRSI
: magnetic resonance spectroscopic imaging
QELT
: quantitative exchange label turnover
SNR
: signal to noise ratio
WM
: white matter

## 1 Introduction

Glucose (Glc) is the primary energy source of the human brain, which utilizes at least 20% of the whole‐body energy consumption to generate adenosine triphosphate (ATP) required for cellular maintenance, neuronal activity and neurotransmitter synthesis [1, 2, 3]. Glucose metabolism plays a critical role in physiological and pathophysiological brain function and an improved understanding of the underlying metabolic model would be beneficial, especially since many wide‐spread neurodegenerative diseases, such as Alzheimer's, epilepsy, psychiatric disorders and brain tumors [4, 5, 6], are characterized by impaired glucose metabolism. Given the critical role, there is a growing clinical interest in developing non‐invasive and robust methods to image glucose metabolism. Recently, Deuterium Metabolic Imaging (DMI) [7, 8, 9, 10, 11, 12, 13] and quantitative exchange label turnover (QELT) [14, 15, 16, 17] have emerged as promising MRS techniques to non‐invasively map the cellular glucose (Glc) uptake and downstream metabolism. Deuterium‐labeled (^2^H‐labeled) glucose, used as a non‐radioactive and harmless tracer, is taken up by brain cells and incorporated into downstream products of the glucose metabolism (i.e., combined glutamate and glutamine (Glx) and lactate (Lac)) and can be dynamically and spatially resolved using DMI. This allows for separating healthy oxidative from pathologic anaerobic pathways represented by oxidatively synthesized Glx and glycolytically synthesized lactate, respectively [7, 11, 13].

The low natural abundance of Deuterium (0.0156%) together with the low sensitivity of the method results in sparse spectra with significantly less signal from contaminating compounds such as water and lipids. Consequently, there is no need for water suppression in DMI and the water resonance can be used as an internal reference for concentration estimation. The sparsity of acquired DMI spectra additionally allows for simpler spectral quantification and a 6.5‐fold lower Larmor frequency makes DMI less sensitive to magnetic field inhomogeneities compared to ^1^H MRSI. Nuclei with a spin quantum number of *I* ≥ 1, as is the case for deuterium *I* = 1, feature a non‐spherically symmetric electric charge distribution. As a result, interactions between the quadrupole moment of the nuclei and electric field gradients generated by surrounding electron clouds become orientation‐dependent. Since the quadrupolar interaction is usually much stronger than any other interactions, it is the dominant relaxation mechanism in nuclei with *I* ≥ 1, e.g., ^2^H, ^17^O, ^39^K resulting in relaxation time constants in the order of milliseconds [18, 19, 20, 21].

As the goal in DMI is a reliable quantification of metabolite concentrations or flux rates [8, 13], knowledge of tissue‐specific relaxation times is crucial. While unlocalized acquisitions represent a simple method to obtain relaxation time values, they do not take into account regional differences and can potentially lead to wrong estimation of metabolite concentrations, especially in pathologies [22, 23], where changes in tissue properties and composition lead to different relaxation times [21]. Additionally, many common diseases are characterized by alterations in glucose uptake and impaired metabolism, including brain tumors [4], neurodegenerative disorders [5] and psychiatric disorders [6], where knowledge of tissue‐specific relaxation times would significantly improve metabolite concentration estimation and potentially provide a better understanding of metabolic variations associated with these diseases. However, to the best of our knowledge and with the exception of deuterated water [24], only unlocalized relaxation constants of ^2^H‐labeled resonances in the human brain have been reported in literature, i.e., glucose (Glc), combined glutamine + glutamate (Glx) or lactate (Lac) [7, 9, 10, 25], which do not reflect potential regional and tissue‐specific differences in relaxation times. There are two main challenges that limit the accurate measurement of relaxation times in DMI: (1) The scan times for conventional MRSI sequences at sufficiently high spatial resolutions to distinguish GM and WM are long (especially when combined with full relaxation conditions in inversion recovery experiments) and single‐voxel MRS alternatives are impeded by the excessive gradient demands at low Larmor frequencies. (2) Measurement of relaxation properties involving tracer administration such as ^2^H‐labeled Glc and Glx are time‐sensitive because metabolite levels remain in a steady state for only a brief period.

To overcome the limitations posed by extended scan durations and insufficient spatial resolution, we propose to combine relaxation time experiments with fast non‐Cartesian readout trajectories to allow spatially resolved measurements of *T*_1_ and *T*_2_ relaxation times. It has been shown in previous studies that the use of spatial‐spectral sampling, i.e., concentric ring trajectories (CRT) enables accelerated scan times up to 100‐ to 300‐fold compared to conventional Cartesian phase‐encoding readout, while being highly SNR‐efficient [26].

In this study, we modified a previously developed 3D ^2^H‐FID‐MRSI sequence using Hamming‐weighted non‐Cartesian concentric ring trajectory sampling [12, 26, 27] with integrated k‐space reordering to asses tissue‐specific ^2^H relaxation times of ^2^H‐labeled resonances, i.e., water, Glc and Glx separately in human GM and WM dominated regions after oral administration of ^2^H‐labeled Glc. This was achieved by adding an inversion pulse prior spin excitation and a spin‐echo pulse after excitation, for measuring *T*_1_ and *T*_2_ relaxation time measurements, respectively. Results were compared with relaxation times derived from unlocalized acquisitions of the same session and with literature values.

## 2 Material and Methods

### 2.1 Sequence Design

**FIGURE 1:**
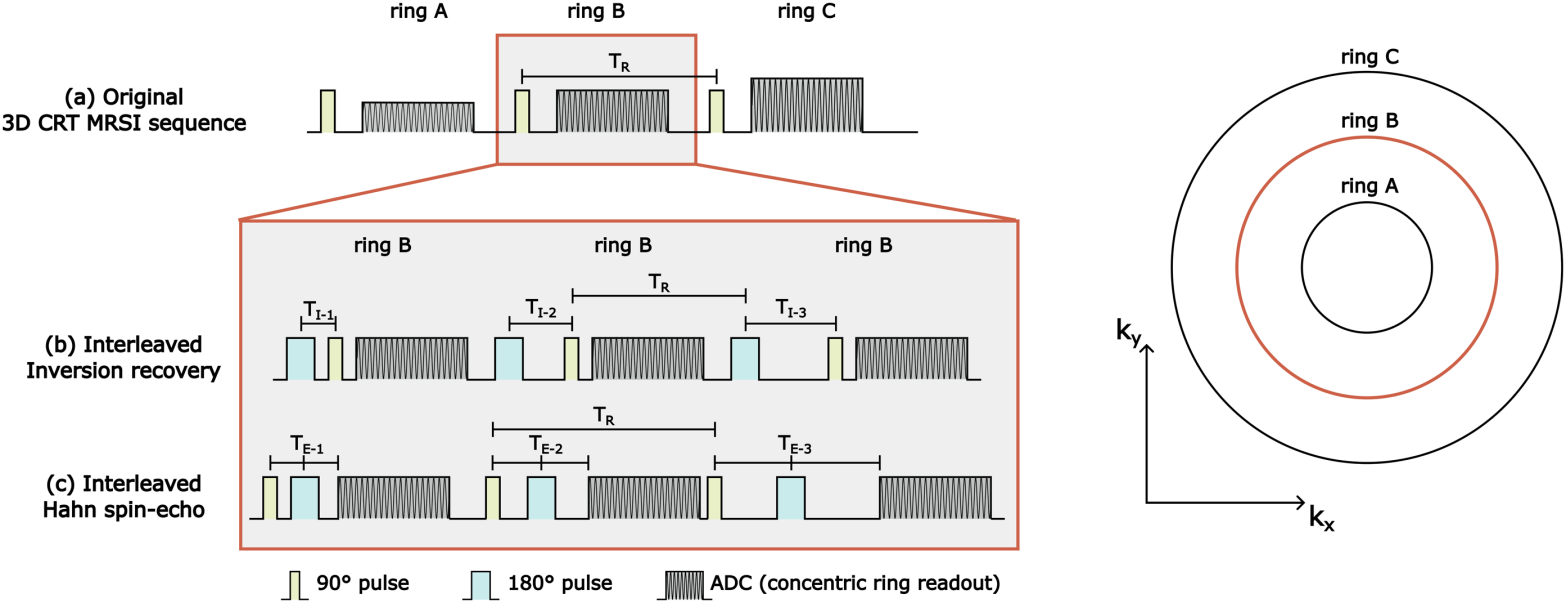
Simplified illustration in 2D of the original 3D FID ^2^H‐MRSI sequence with three representative concentric ring trajectories A–C (a). To allow relaxation time measurements inversion recovery (b) and Hahn spin‐echo (c) acquisition schemes were implemented with specific k‐space reordering. Instead of sampling all ring trajectories consecutively for a specific *T*_I_/*T*_E_, we first loop over all different *T*_I_/*T*_E_s (i.e., *T*_I‐1_, *T*_I‐2_, … and *T*_E‐1_, *T*_E‐2_, …) before increasing the radius. This ensures that different *T*_I_/*T*_E_s are acquired in a short period of time to avoid bias from concentration changes of deuterated compounds. To further minimize this effect k‐space, is sampled from k‐space center outwards. Glc and Glx levels can additionally be monitored by temporally interleaved unlocalized FID acquisitions (approximately every min).

A previously developed 3D ^2^H‐FID‐MRSI sequence using Hamming‐weighted non‐Cartesian CRT readout [12, 26, 27] was modified to allow spatially resolved dynamic assessments of relaxation time constants (*T*_1_ or *T*_2_) within single scans. Inversion recovery and Hahn spin‐echo acquisitions schemes were implemented in the sequence with k‐space reordering of the ring trajectories. Instead of sampling each 4D k‐space (three spatial and one spectral dimension) for each inversion/echo time (*T*_I_/*T*_E_) separately, k‐space encoding was reordered: each ring trajectory was sampled repeatedly with variable inversion or echo times, simplified as encoding a 5D k‐space (three spatial, one spectral, one inversion/echo dimension), see Figure 1. This was done to ensure minimal and identical delay between different inversion/echo times for each *k*‐space point (*T*_1_: 6.6 s, *T*_2_: 2.8 s), where metabolic concentration changes are negligible. Additionally, the 3D *k*‐space was sampled from “inside out” (i.e. starting in the center and moving outwards) to acquire the majority of the signal contribution in the beginning of the measurement. To monitor levels of ^2^H Glc and ^2^H Glx throughout the measurement, unlocalized FID acquisitions were additionally interleaved after every 70^th^ *T*_R_ (approximately every min).

### 2.2 Measurement Protocol

All measurements were performed on an experimental whole‐body 7 T MR system (MAGNETOM‐dot‐Plus; Siemens Healthineers, Erlangen, Germany) using a ^2^H/^1^H dual‐tuned quadrature bird‐cage head coil (Stark Contrast MRI, Erlangen, Germany).

#### 2.2.1 Phantom

The modified 3D ^2^H‐FID‐MRSI sequence was validated on a spectroscopic water phantom (diameter of 160 mm containing water with added lithium lactate: 96 mM and sodium acetate: 100 mM) from Siemens. Measured relaxation time values of the natural abundance ^2^H water were compared with values obtained using an unlocalized sequence as a reference gold standard, with matched *T*_I_ and *T*_E_ parameters (CRT: voxel volume = 1.96 mL, matrix size: 16 × 16 × 15, FOV: 200 × 200 × 170 mm^3^; CRT and unlocalized sequence: *T*_R_ = 1500 ms; six *T*_I_s: 5‐1500 ms; seven *T*_E_s: 10‐1000 ms).

#### 2.2.2 In Vivo

**FIGURE 2:**
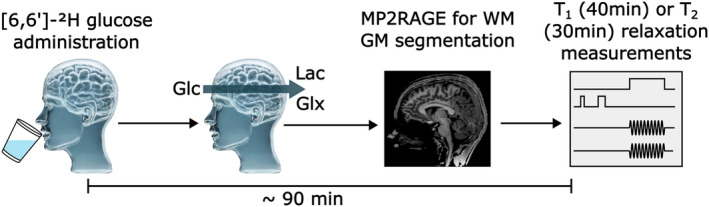
Illustration of in vivo measurement protocol, which included anatomical MP2RAGE scans for segmentation of WM/GM dominant regions. Relaxation time measurements were performed ~90 min after oral administration of [6,6′]‐^2^H glucose administration in the steady‐state.

Thirteen healthy volunteers (age: 27 ± 5; BMI 23 ± 3; 9 m/4 f) without a history of neurological, psychiatric, or metabolic diseases were scanned after written informed consent was obtained. The study was approved by the local ethics committee of the Medical University of Vienna according to the guidelines of the Declaration of Helsinki. Ten volunteers underwent oral ^2^H Glc administration before relaxation time scans. Two of them were rescanned without ^2^H Glc administration for natural abundance ^2^H measurements and three others were scanned only without ^2^H Glc administration. Scans were performed in the morning after overnight fasting and during presumed steady state, i.e., ~90 min after oral ^2^H‐labeled Glc administration (0.8 g/kg body weight, [6,6′]‐^2^H‐Glc ≥99% purity, Cambridge Isotopes) dissolved in ~200 mL water, see Figure 2. Before the actual relaxation time measurements, the study protocol included automated alignment localizer followed by *B*_0_ shimming using the standard shimming routine supplied by the vendor. Flip angle calibration was performed for each volunteer using unlocalized pulse‐acquire B_1_ estimation (*T*_R_ = 1500 ms, *T*_E_ = 0.35 ms, 20 steps, *U*_Ref_ = 20–440 V) to find the optimal reference voltage to approximate 90° excitation and 180° inversion/refocusing flip angles. Additionally, anatomical MP2RAGE acquisitions (FOV: 165 × 220 × 220 mm^3^, matrix: 144 × 192 × 192, *T*_R_ = 3930 ms, *T*_I1_ = 850 ms, *T*_I2_ = 3400 ms, *T*_E_ = 3.28 ms, *T*_A_ = 9: 29 min) were performed for GM and WM tissue segmentation. 3D CRT ^2^H MRSI measurements (either *T*_1_ or *T*_2_) were performed ~90 min after Glc intake using the following parameters: FOV = 200 × 200 × 192 mm; matrix size = 22 × 22 × 21; nominal isotropic volume = 0.75 mL; 148/96 samples (*T*_1_/*T*_2_); *T*_R‐T1/T2_ = 500/400 ms; six *T*_I_s: 5–500 ms; eight *T*_E_s: 6–100 ms, *T*_A‐T1/T2_ = 40/30 min. Five subjects were scanned without Glc administration to measure relaxation times of natural abundance ^2^H water (*T*_R‐T1/T2_ = 900/400 ms; five *T*_I_s: 5–900 ms; six *T*_E_s: 6–60 ms). For detailed information about sequence parameters, see Table S1 for minimum reporting standards [28].

### 2.3 Data Reconstruction

Data was reconstructed using in‐house developed post‐processing pipelines (MATLAB R2021, LCModel v6.3, Python v3.10) including non‐Cartesian three‐dimensional discrete Fourier Transformation without density compensation [12, 26, 27], since we matched the target filter with the acquired weights. Calculation of tissue‐specific relaxation times was based on high‐resolution *T*_1_‐weighted anatomical 3D images (MP2RAGE), which were segmented in GM and WM regions using FAST [29]. High resolution images were down sampled in *k*‐space to match the spatial resolution of the MRSI data, while considering effects of the point spread function and partial volume contamination of increased voxel size. Due to inherently low SNR per voxel, *T*_1_ and *T*_2_ mapping of the in vivo relaxation times over the whole brain was not feasible. Therefore, spectral data was averaged separately for GM and WM dominated regions including prior phasing to account for spatially different phase and frequency, followed by spectral fitting. To estimate GM and WM dominated voxels an initial threshold of 40% WM (GM) was used with the additional, requirement of voxels exhibiting at least 50% more WM (GM) content than GM (WM) content and vice versa.

### 2.4 Spectral Fitting

Spectral fitting was performed voxel wise for the phantom and over regionally averaged data for in vivo measurements in the frequency domain using LCModel. No data points were excluded using a strict CRLB threshold. For in vivo measurements a custom‐built basis set was simulated [30, 31, 32] featuring ^2^H resonances of water (4.8 ppm), Glc (3.9 ppm), and Glx (2.4 ppm), considering different acquisitions delays to match first‐order phase (*T*_1_: 2 ms, *T*_2_: 0 ms). For accurate fitting of acquired data of *T*_1_ relaxation time measurements the basis set unconventionally included corresponding metabolite peaks with 180° phase offset, which were required due to significantly different *T*_1_ relaxation time constants between water, Glc and Glx. Depending on the inversion time *T*_I_ and based on estimated relaxation times of the metabolites, corresponding basis sets were selected for spectral fitting. Basis set used for phantom measurements and scans without Glc administrations featured only the peak of ^2^H water.

### 2.5 Relaxation Time Fitting and Statistical Analysis

Exponential fitting of relaxation times was done assuming a three parameter ($M\left(\mathrm{TI}\right)=C_{1}\left(1-C_{2}*e^{-\mathrm{TI}/T_{1}}\right)$) and two parameter ($M\left(\mathrm{TE}\right)=C_{1}*e^{-\mathrm{TE}/T_{2}}$) fit for *T*_1_ and *T*_2_ experiments, respectively. Unlocalized water *T*_2_ relaxation times were obtained using a bi‐exponential fit [7, 10]. To account for different compartments in the human brain, longer relaxation times of the natural abundance water for extracellular fluid (CSF) and shorter relaxation times for intracellular fluid (GM and WM): $M_{\mathrm{bi}-\exp}\left(\mathrm{TE}\right)=C_{1}\left(f_{\mathrm{CSF}}*e^{-\mathrm{TE}/T_{2}^{\text{long}}}+f_{\mathrm{GM}+\mathrm{WM}}*e^{-\mathrm{TE}/T_{2}^{\text{short}}}\right)$ were assumed. Tissue fractions (f_CSF_ : f_GM + WM_) were estimated based on MP2RAGE anatomical images over the whole brain for each volunteer.

To assess significant differences between GM, WM, and unlocalized relaxation time constants, paired *t*‐test was applied, due to small sample sizes [33], with a statistical significance threshold of *p* < 0.05. Relaxation time exponential fitting and statistical analysis were performed using Python (v3.10, curve_fit function from scipy.optimize and scipy.stats). Monitored Glc and Glx levels, acquired with global unlocalized FID acquisitions over time during in vivo measurements, were analyzed using jMRUI and AMARES [34] for spectral fitting. To analyze the temporal stability of metabolite concentrations, coefficients of variation (COV) were calculated throughout the whole experiment averaged over all volunteers.

### 2.6 Synthetic Data

To compare the proposed k‐space reordering sampling scheme with conventional consecutive k‐space sampling two noise‐less high‐resolution brain MRSI datasets (220 × 220 × 210 voxels, 96 spectral points, ^2^H water resonance only) were simulated with two synthetic compartments (synthetic gray matter (sGM) and white matter (sWM)) and distinct relaxation times: *T*_1_^sGM/sWM^ = 350/310 ms (five inversion times: 5–900 ms) and *T*_2_^sGM/sWM^ = 35/25 ms, (seven echo times: 6–60 ms)). To account for point spread function effects and partial volume contamination due to Hamming weighting, datasets were down‐sampled to match the matrix size of the measurement protocol (i.e. matrix size: 22 × 22 × 21). Masks for segmentation of sGM and sWM voxels were created in a similar fashion. Additionally, linearly decreasing and increasing (±35%) metabolite levels were simulated as a linear 3D spherical k‐space weighting function for k‐space reordered sampling and by linearly weighting inversion/echo time separately for conventional consecutive k‐space sampling, see Figure S1. Spectral fitting and relaxation time estimation of the synthetic data was performed voxel wise using the same post processing pipeline as for in vivo data, followed by regional averaging over sGM and sWM dominated regions using downsampled masks (threshold > 60%). To assess the robustness of our proposed sequence against changes in metabolite concentration, we compared relaxation times of k‐space reordered and conventional consecutive k‐space sampling approaches with known simulated tissue‐specific relaxation times (considered the gold standard).

## 3 Results

### 3.1 Phantom Measurements

^2^H water relaxation times calculated from phantom data were consistent between CRT‐based 3D‐^2^H‐MRSI and unlocalized acquisitions (*T*_1_^CRT averaged^ = 428 ± 4 ms, *T*_1_^unlocalized^ = 428 ± 3 ms; *T*_2_^CRT averaged^ = 422 ± 8 ms, *T*_2_^unlocalized^ = 442 ± 4 ms). Additionally, voxel wise SNR was sufficiently high to calculate relaxation times voxel wise in phantom measurements (*T*_1_^CRT voxel wise^ = 424 ± 32 ms, *T*_2_^CRT voxel wise^ = 464 ± 24 ms).

### 3.2 ^2^H Glc and ^2^H Glx Resonances

**FIGURE 3:**
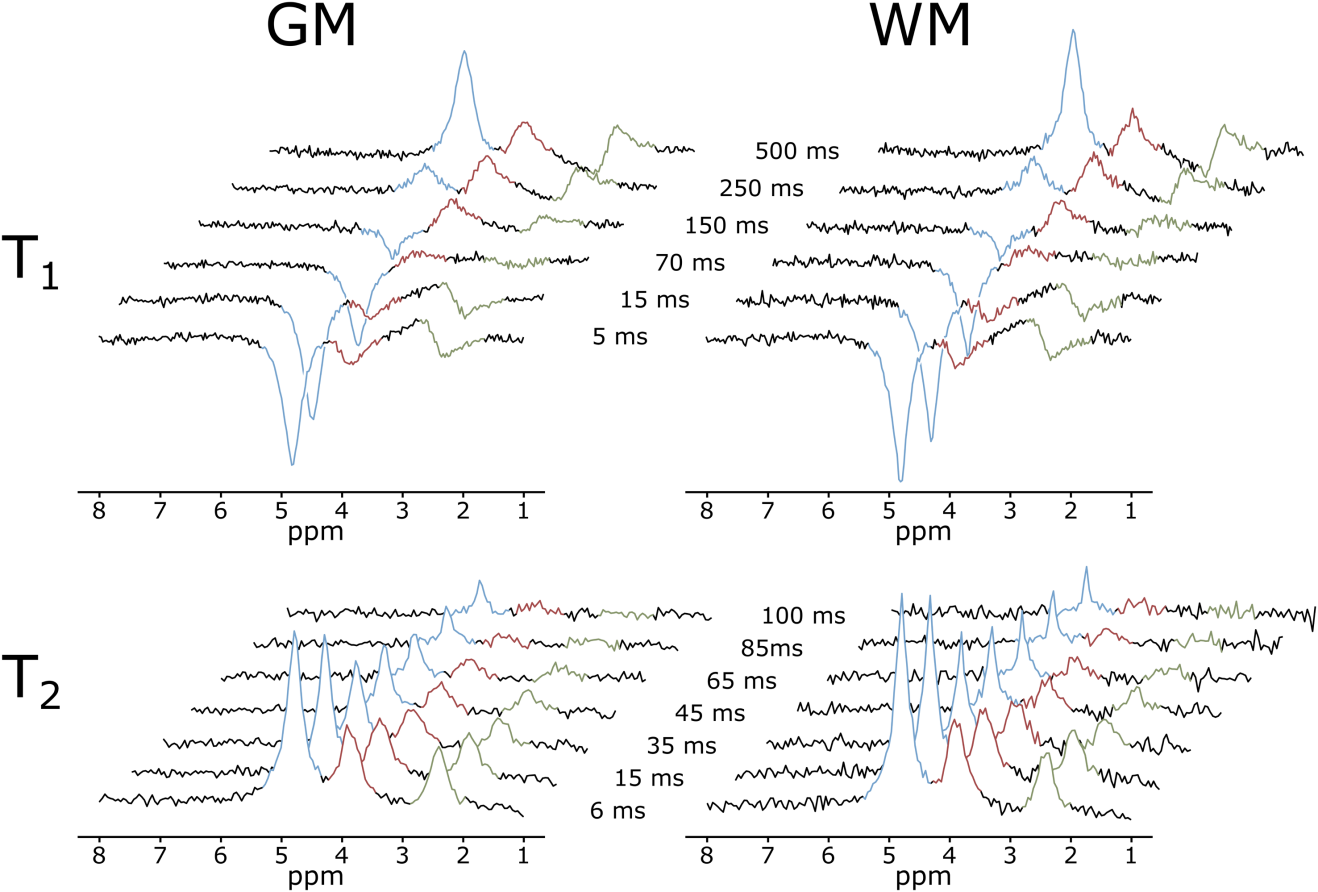
Inversion recovery (*T*_1,_ top) and Hahn spin‐echo (*T*_2,_ bottom) spectra series of gray matter (GM, left) and white matter (WM, right) dominated regions, of the human brain from two representative volunteers. Spectra represent deuterium resonances of the natural abundant water (blue peak), Glc (red peak) and Glx (green peak) acquired during *T*_1_ and *T*_2_ relaxation measurements using CRT based 3D ^2^H‐MRSI acquisitions. Signal evolution is illustrated for increasing *T*_I_ (5–50 ms)/*T*_E_ (6–100 ms). Spectra were averaged over GM (T_1_/T_2_: 434/398 mL) and WM (T_1_/T_2_: 211/243 mL) dominated regions due to too low voxel wise SNR. Relaxation dynamics are clearly visible with Glc featuring the shortest relaxation time followed by Glx and water.

In vivo relaxation time measurements of ^2^H Glc and ^2^H Glx were employed on average 95 ± 8 min after administration of ^2^H‐labeled Glc during presumed steady state with a scan time of 40 and 30 min for *T*_1_ and *T*_2_ relaxation time experiments, respectively. The measurement protocol was extended for two volunteers and included assessment of both relaxation times during one session, and to avoid bias of potentially decreasing metabolite levels towards the end of the experiment, the order of *T*_1_ and *T*_2_ relaxation time measurements was alternated between both volunteers. Representative averaged high‐SNR spectra series illustrating the signal evolution for increasing *T*_I_/*T*_E_ times are shown in Figure 3. The CRLBs of averaged spectra were below 30% except for long *T*_E_ in case of *T*_2_ measurements. SNR, FWHM and number of averaged voxels are listed in Table S2.

**FIGURE 4:**
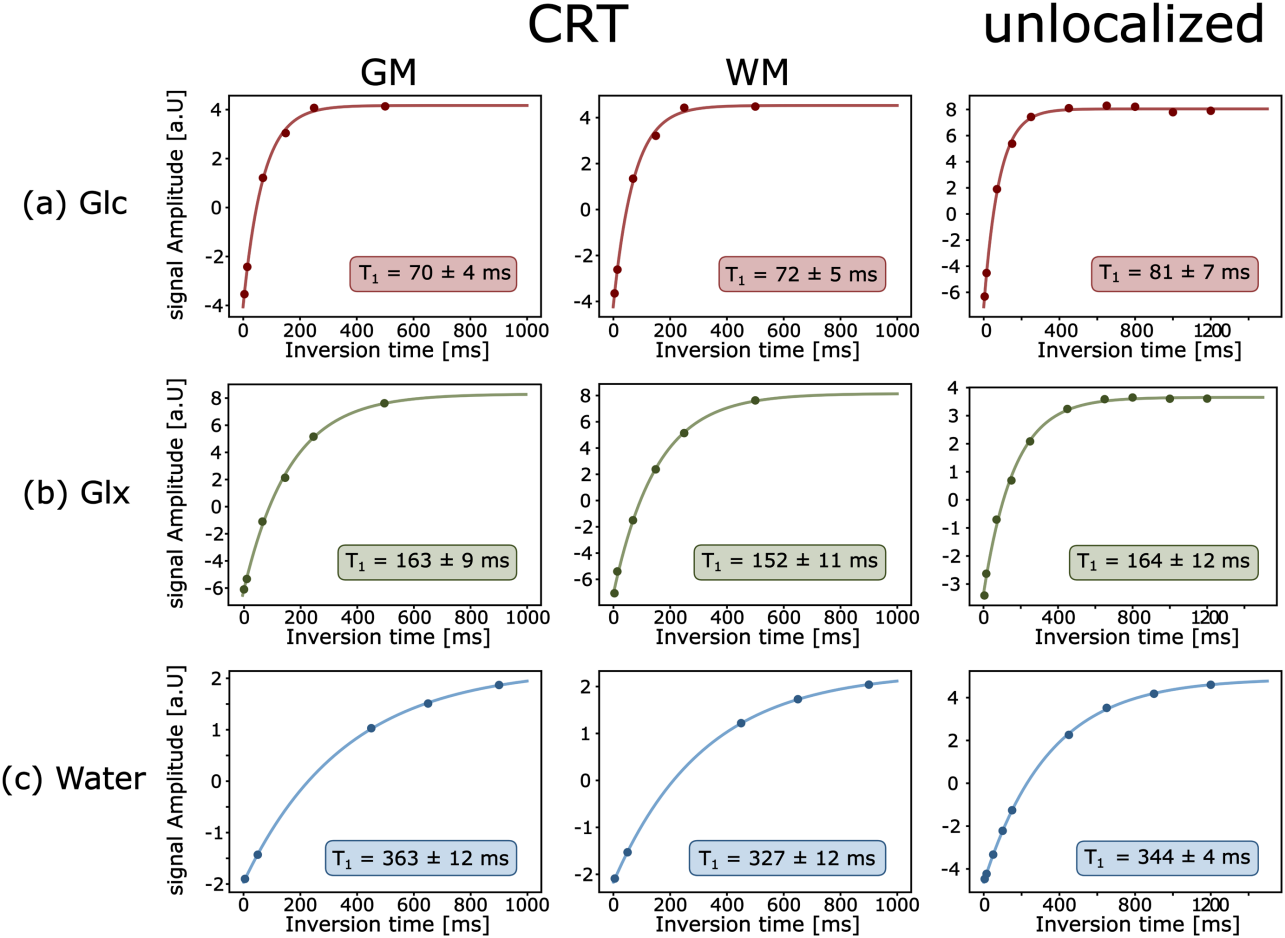
Representative exponential fits for Inversion recovery experiments to quantify *T*_1_ relaxation times of glucose (Glc, red), combined glutamate+glutamine (Glx, green) and water (blue) from one healthy volunteer. Scans were performed after (a + b) oral administration of deuterated glucose and without ^2^H Glc administration (c). Data was acquired using a newly developed 3D ^2^H‐MRSI sequence using concentric ring trajectory readout with specific reordering of the k‐space, which enabled measurements of relaxation times separately in GM and WM dominated regions and compared to unlocalized scans.

**FIGURE 5:**
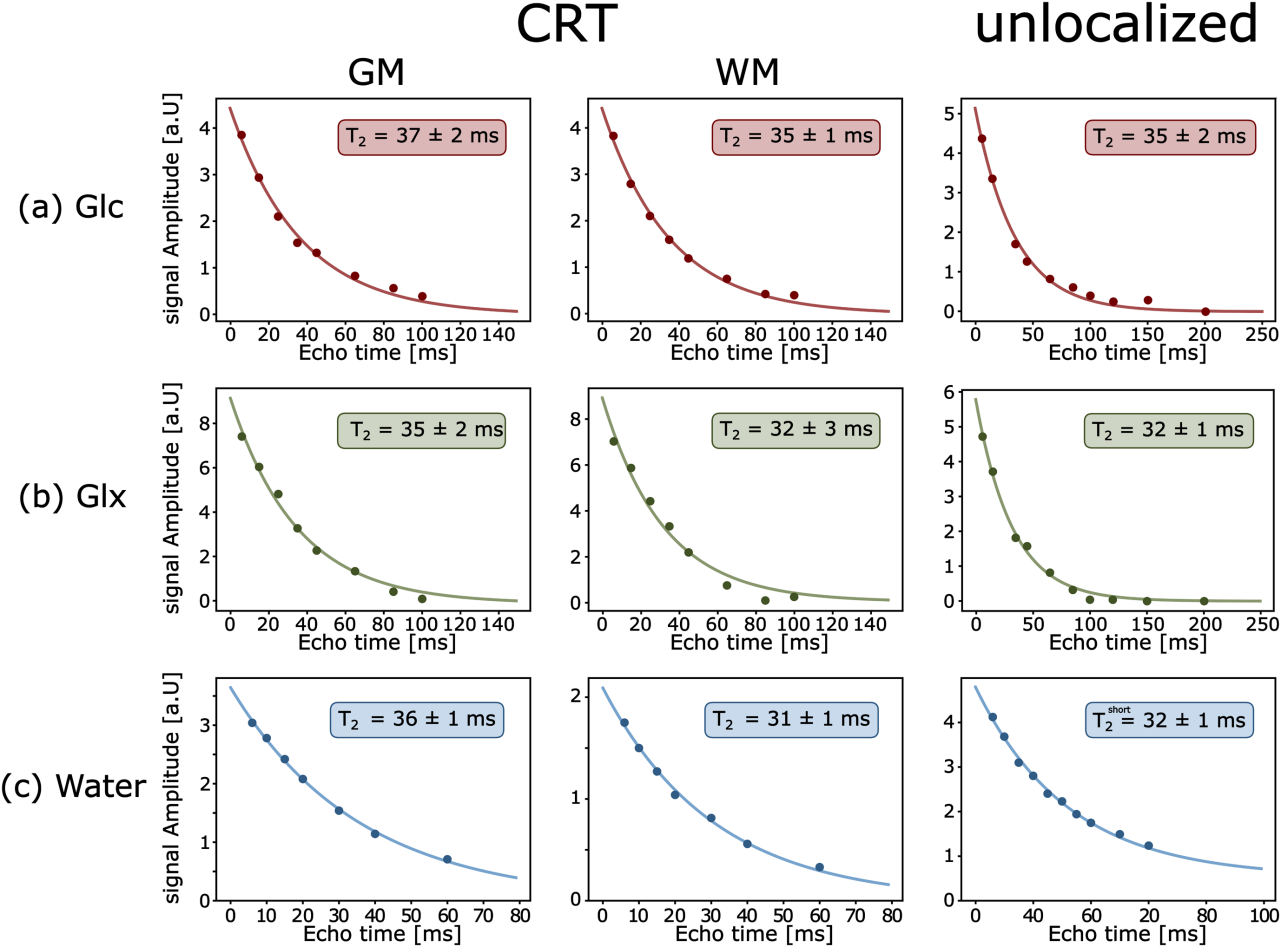
Representative exponential fits for Hahn spin‐echo experiments to quantify *T*_2_ relaxation times of glucose (Glc red), combined glutamate+glutamine (Glx, green) and water (blue) from one healthy volunteer. Scans were performed after (a + b) oral administration of deuterated Glc and without ^2^H Glc administration (c). Data was acquired using a newly developed 3D ^2^H‐MRSI sequence using concentric ring trajectory readout with specific reordering of the k‐space, which enabled measurements of relaxation times separately in GM and WM dominated regions and compared to unlocalized scans, with *T*_2_ relaxation constants of unlocalized water scans fitted using a bi‐exponential fit.

Similar tissue‐specific *T*_1_ relaxation times were estimated for ^2^H Glc (*T*_1_^GM/WM^ = 70 ± 5/73 ± 4 ms, *p* = 0.13) and ^2^H Glx (*T*_1_^GM/WM^ = 172 ± 15/172 ± 12 ms, *p* = 1.00). Furthermore, no regional differences were observed for derived *T*_2_ relaxation times for ^2^H Glc (*T*_2_^GM/WM^ = 36 ± 1/34 ± 2 ms, *p* = 0.24), while tissue‐specific differences were found for *T*_2_^2^H Glx (*T*_2_^GM/WM^ = 37 ± 2/35 ± 2 ms, *p* = 0.02). Individual results of all volunteers are summarized in Table 1 and representative exponential fits of one volunteer are shown in Figures 4a,b and 5a,b.

**TABLE 1:** Relaxation times [ms] of deuterated resonances Glc and Glx in the human were measured in 10 healthy volunteers after oral administration of [6,6′]‐^2^H Glc: *T*_1_ (top) and *T*_2_ (bottom). Relaxation times of GM and WM matter dominated regions acquired using CRT based 3D ^2^H‐MRSI are compared to relaxation times acquired with unlocalized scans. No statistical differences were found between GM and WM dominated regions, except for *T*_2_ relaxation time of Glx.

		Glc			Glx		
			CRT			CRT	
	Subject	Unlocalized	Gray matter	White matter	Unlocalized	Gray matter	White matter
T_1_ [ms]	1	74 ± 7	66 ± 8	69 ± 8	155 ± 12	172 ± 12	183 ± 11
	2	76 ± 7	64 ± 8	69 ± 4	164 ± 12	174 ± 8	185 ± 8
	3	73 ± 6	70 ± 3	75 ± 3	185 ± 18	196 ± 16	165 ± 24
	4	85 ± 7	73 ± 8	80 ± 4	159 ± 16	185 ± 22	177 ± 18
	5	81 ± 7	70 ± 4	72 ± 5	164 ± 12	163 ± 9	152 ± 11
	6	85 ± 9	79 ± 7	72 ± 6	175 ± 12	153 ± 8	164 ± 6
	7	84 ± 10	66 ± 3	75 ± 6	154 ± 7	161 ± 14	178 ± 20
	Mean ± SD	80 ± 5	70 ± 5	73 ± 4	165 ± 11	172 ± 15	172 ± 12
T_2_ [ms]	8	32 ± 2	35 ± 1	34 ± 1	31 ± 2	34 ± 2	33 ± 2
	9	35 ± 2	37 ± 2	35 ± 1	32 ± 1	35 ± 2	32 ± 3
	10	33 ± 2	37 ± 1	34 ± 2	31 ± 4	38 ± 2	35 ± 3
	6	34 ± 2	35 ± 2	37 ± 3	36 ± 2	38 ± 2	37 ± 3
	7	37 ± 2	34 ± 1	32 ± 1	36 ± 3	40 ± 4	36 ± 2
	Mean ± SD	34 ± 2	36 ± 1	34 ± 2	33 ± 3	37 ± 2*	35 ± 2

Differences between CRT based 3D ^2^H‐MRSI and unlocalized acquisitions were observed for Glc (*p*_GM/WM_^T1^ = 0.002/0.01) and in GM for Glx (*p*_GM_^T2^ = 0.01).

Temporally interleaved unlocalized FID acquisitions allowed for monitoring stability of Glc and Glx levels throughout the entire measurement protocol. Figure S2 shows averaged time courses of Glc and Glx concentrations across all volunteers for *T*_1_ and *T*_2_ relaxation time experiments, respectively. Time evaluation of the FID data revealed relatively stable global Glc and Glx levels throughout the measurements with a COV of 7 ± 3% and 6 ± 1% across all volunteers, respectively.

### 3.3 Natural Abundance ^2^H Water

Measurements to estimate *T*_1_ and *T*_2_ relaxation times of the natural abundant ^2^H water resonance could be acquired in a single session as no oral Glc administration was required and were therefore less time sensitive. For averaged high SNR (SNR > 40) spectra CRLBs were below 5%; SNR, FWHM and number of averaged voxels are listed in Table S2. Significant differences in relaxation times were found between GM and WM dominated regions for *T*_1_ relaxation constants (*T*_1_^GM^ = 358 ± 21 ms, *T*_1_^WM^ = 328 ± 12 ms, *p* = 0.01), while no regional differences were found for *T*_2_ relaxation times (*T*_2_^GM^ = 36 ± 2 ms, *T*_2_^WM^ = 34 ± 2 ms, *p* = 0.08). Values were overall in good agreement with results obtained using unlocalized scans (*T*_1_^unlocalized^ = 335 ± 6 ms; *T*_2_^unlocalized^ = 31 ± 2 ms), while differences between GM and unlocalized acquisitions were observed for *T*_2_^GM^ (*p* = 0.0004). Individual values of all volunteers are listed in Table 2 and exponential fits of relaxation time constants of one volunteer are shown in Figures 4c and 5c.

**TABLE 2:** Relaxation times [ms] of the natural abundance water in the human brain were measured in five healthy volunteers without Glc administration. Relaxation times of GM and WM dominated regions acquired using CRT based 3D ^2^H‐MRSI are compared to relaxation times acquired with unlocalized scans. Significant difference between GM and WM matter dominated regions was observed for T_1_ relaxation.

		T_1_ [ms]			T_2_ [ms]		
			CRT			CRT	
	Subject	Unlocalized	Gray matter	White matter	Unlocalized	Gray matter	White matter
Water	1	334 ± 5	363 ± 12	327 ± 12	32 ± 1	36 ± 1	31 ± 1
	2	—	383 ± 7	348 ± 14	—	39 ± 1	37 ± 2
	11	343 ± 4	364 ± 15	318 ± 7	28 ± 1	33 ± 1	33 ± 0
	12	331 ± 6	326 ± 12	323 ± 3	31 ± 1	35 ± 1	33 ± 1
	13	330 ± 4	356 ± 12	322 ± 2	31 ± 2	35 ± 1	34 ± 1
	Mean ± SD	335 ± 6	358 ± 21*	328 ± 12	31 ± 2	36 ± 2	34 ± 2

### 3.4 Synthetic Data

Relaxation time fits of the gold standard (GS), k‐space reordered and conventional consecutive k‐space sampling are shown in Figure S3. Relaxation times between the GS (*T*_1_^sGM/sWM^ = 347 ± 5/310 ± 6 ms; *T*_2_^sGM/sWM^ = 34 ± 1/27 ± 1 ms) and k‐space reordered approach (increase: *T*_1_^sGM/sWM^ = 347 ± 5/309 ± 7 ms; *T*_2_^sGM/sWM^ = 35 ± 1/27 ± 1 ms; decrease: *T*_1_^sGM/sWM^ = 347 ± 5/312 ± 6 ms; *T*_2_^sGM/sWM^ = 34 ± 1/27 ± 1 ms) were comparable, yielding a minimal error of 0–3%, whereas relaxation times obtained with the conventional approach (increase: *T*_1_^sGM/sWM^ = 501 ± 9/442 ± 10 ms; *T*_2_^sGM/sWM^ = 45 ± 2/34 ± 2 ms; decrease: *T*_1_^sGM/sWM^ = 215 ± 3/193 ± 4 ms; *T*_2_^sGM/sWM^ = 25 ± 1/21 ± 1 ms) were on average 38–44% and 22–32% different for *T*_1_ and *T*_2_ relaxation times, respectively.

## 4 Discussion

In this study we assessed tissue‐specific *T*_1_ and *T*_2_ relaxation times of ^2^H‐labeled metabolites, i.e., water, glucose (Glc) and glutamate+glutamine (Glx) in the human brain at 7 T using a novel 3D ^2^H‐MRSI sequence. Inversion‐recovery and Hahn spin‐echo acquisition schemes were implemented with variable delays and combined with a Hamming‐weighted non‐Cartesian CRT readout including k‐space reordering of the ring trajectories to perform 3D relaxation time measurements within single scans with a sub‐milliliter nominal isotropic resolution.

Sequence functionality was successfully validated as *T*_1_ and *T*_2_ relaxation times derived from phantom measurements were consistent between CRT based 3D ^2^H‐MRSI and unlocalized acquisitions. Additionally, obtained values were in good agreement with literature [10] even for voxel wise relaxation time fitting, although higher SD and COVs were observed due to decreased SNR, reducing the fit accuracy, especially for exponentially decaying *T*_2_ relaxation estimation.

Voxel wise mapping of the relaxation times was not feasible for in vivo measurements due to inherently lower SNR. However, spectral averaging over GM and WM dominated regions increased the SNR substantially and allowed for reliable spectral fitting and tissue‐specific relaxation time determinations of ^2^H‐labeled resonances. Determination of relaxation times in CSF regions using CRT based 3D ^2^H‐MRSI was not feasible because too few voxels met the threshold criteria for regional averaging leading to insufficiently low SNR for reliable spectral fitting.

Averaged data points featured low CRLBs (^2^H water < 5% and ^2^Glc/^2^Glx < 30%), except low SNR data points, which correspond to long echo times of *T*_2_ measurements. Since data points of long echo times are essential for accurate relaxation time fitting, we decided not to exclude any data point. Additionally using relative CRLBs to estimate the standard deviation and applying a strict threshold for excluding data points when dealing with low SNR data is not always recommended [35, 36].

Measured tissue‐specific *T*_1_ and *T*_2_ values of ^2^H Glc and ^2^H Glx acquired using CRT based 3D ^2^H‐MRSI were not significantly different between GM and WM dominated regions, except for *T*_2_^Glx^ values. Although the nominal spatial resolution increased compared to the majority of whole‐brain DMI studies, it is still a major limitation of the applied method, leading to an imperfect point spread function and significant partial volume contamination. Given the small sample size of this study (*n*_T1_ = 7, *n*_T2_ = 5), this could presumably explain that either small differences could not be reliably detected or no substantial difference between GM and WM dominated regions are present. Given the high costs associated with measurements using ^2^H Glc, and our findings indicating potential variations in relaxation times between different brain tissues are too small to significantly bias the estimation of metabolite concentrations, increasing the number of subjects would not be reasonable. While, similar *T*_2_* relaxation values were reported between GM and WM for other nuclei with *I* > 1/2, which are also dominated by quadrupolar relaxation mechanism, (in rat brain for ^17^O at 16T [37] and in vivo for ^23^Na at 7 T [38, 39]), it should be noted that despite commonality of quadrupolar relaxation among ^2^H, ^17^O and ^23^Na, other processes such as the chemical structure in which the nuclei are located may play a significant role and regional difference of ^2^H relaxation times cannot completely be ruled out.

Regionally specific relaxation times obtained with CRT based 3D ^2^H‐MRSI were overall consistent with relaxation times derived from unlocalized pulse‐acquire based data, while significant differences were observed for Glc (*T*_1_^GM/WM^) and for Glx (*T*_2_^GM^). Unlocalized *T*_1_ relaxation time values are slightly longer (10%) compared to literature values reported at the same field strength [10]. This can possibly be caused by differences in the fitting model, (number of inversion times) or coil setup (volume coil vs. array coil) leading to different sensitivities.

The unlocalized *T*_2_ relaxation times of ^2^H Glc and ^2^H Glx in this study, acquired at 7 T, range in between reported values acquired at 4 T and 11.7T^7^, which is in good agreement with literature, supporting the expectation of decreasing *T*_2_ relaxation time constants with increasing magnetic field strength [25, 40]. However, longer *T*_2_ relaxation times for Glx and Glc were reported at 7 T (*T*_2_^Glc/Glx^ = 44 ± 4/53 ± 2 ms) compared to our data or literature values acquired at 4T [7].

Relaxation constants of ^2^H‐labeled Glc and downstream products (Glx) were obtained after oral administration of ^2^H Glc, where Glc and Glx levels are assumed to be stable. Comparable studies using similar levels of oral [6,6′]‐^2^H‐Glc administration observed stable Glc and Glx levels after approximately 90 min and the plateau phase maintained relatively stable for another 60–80 min [7, 9]. Stable metabolite concentrations are crucial, in general, to ensure accurate assessment of relaxation times. Therefore, metabolite levels (Glc and Glx) were monitored during the entire relaxation time measurements by temporally interleaving unlocalized FID acquisitions, which could be theoretically used to correct very strong signal variations during post‐processing. Low coefficients of variation (COV < 7%) of unlocalized metabolite levels confirmed that Glc and Glx levels remained relatively stable throughout the entire measurement, with only a marginal overall decreasing and increasing trend visible for averaged mean Glc and Glx metabolite concentrations, respectively. Specific reordering of the k‐space trajectories to minimize time delays between the acquisitions of all inversion/echo times for each k‐space point, combined with an ‘inside‐out’ sampling scheme of the 3D k‐space that acquires lower frequencies at the k‐space center at the beginning of the measurement, should further minimize the effects of potentially decreasing metabolite concentrations towards the end of the scan, which could otherwise lead to additional k‐space weighting and increased spatial blurring. Simulations demonstrated that our proposed method of encoding the k‐space in a reordered fashion is unaffected by fluctuations in metabolite levels and accurately measures relaxation times even when stable conditions are not maintained. Our method produced values consistent with the gold standard (constant metabolite levels), showing only minor deviations of 0–3%, despite simulated ±35% decrease/increase in metabolite levels during the measurement period. In contrast, conventional consecutive encoding of the k‐space separately for each T_I_/T_E_ without reordering resulted in relaxation times that differed by 22–44% from the gold standard.

*T*_1_ relaxation times of natural abundant ^2^H water were significantly different between GM and WM dominated regions, while no differences were found for *T*_2_ relaxation constants. This is consistent with theoretical predictions [21] regarding the dependence of relaxation time on the rotational correlation time of water molecules, which varies between GM and WM tissues and is linked to their structural, molecular and cellular differences [21, 41, 42]. Lower water content [41] in WM together with a more organized microstructure [43] leads to shorter relaxation times characterized by a longer rotational correlation time, whereas the less structured and rigid environment in GM leads to shorter correlation times and therefore longer relaxation times.

Measured *T*_1_ relaxation constants of natural abundant ^2^H water in GM and WM dominated regions were also in good agreement with tissue‐specific values reported by Cocking et al. [24] (*T*_1_^GM^ = 320 ± 50 ms, *T*_1_^WM^ = 290 ± 30 ms), which were acquired using a multi‐echo gradient‐echo ^2^H MRI sequence with higher spatial resolution after heavy water loading. Furthermore, tissue‐specific *T*_2_ values calculated in our study were slightly longer than *T*_2_* values reported by the same group (*T*_2_^* GM^ = 32 ± 1 ms, *T*_2_^* WM^ = 30 ± 1 ms). Unlocalized acquisitions were analyzed using bi‐exponential fit for *T*_2_ relaxation to account for different compartments in the human brain [7, 10] (extracellular fluid: slower relaxing cerebral spine fluid (CSF) and intracellular fluid: faster relaxing combined GM and WM). It should be noted that in contrast to *T*_2_^short^, *T*_2_^long^ values did not converge. A possible explanation could be that our coil setup, which uses a volume coil instead of array coils as often used by other groups, is less sensitive to CSF on the border of the brain resulting in reduced CSF contributions to the overall signal, which are difficult to fit. This is also supported by the fact that contaminating lipid signals from the skull are below noise level in our data, whereas other groups using array coils, which are more sensitive around the border of the brain, reported appearance of lipid contributions in their DMI signals [7, 9, 10].

Knowledge of correct relaxation times is crucial for estimating metabolic concentrations and kinetics of energy metabolites, i.e., Glc, Glx, representing potential biomarkers of many severe brain pathologies featuring regional differences in brain Glc metabolism [4, 5, 6]. Yet, to the best of our knowledge only global values of ^2^H‐labeled resonances have been reported and studies only focused on relaxation times of the human brain. Unlocalized FID relaxation measurements are easy to implement, but for tissues located deep within the human body, e.g., tumor tissue, liver or kidney, localized approaches would be beneficial. Our developed 3D CRT based relaxation sequence features high flexibility and can in principle be applied to any other part of the human body to measure relaxation times of different tissues and can be employed to other MR sensitive nuclei as well, making it a versatile and highly flexible sequence which could help to improve the accuracy of relaxation time measurements. Depending on the SNR relaxation time fitting can be applied either voxel wise or after regional averaging over the tissue of interest. However, voxel wise mapping of relaxation times is challenging due to inherently low SNR in DMI, which is one of the main limitations of our method, especially for small regions of interest with limited number of voxels for averaging in the spectral domain, making it challenging to obtain reliable relaxation time values from these areas. Implementation of advanced denoising methods [44, 45], compressed sensing reconstruction approaches using k‐t undersampling [46, 47, 48], or deep learning techniques [49] could significantly enhance the signal‐to‐noise ratio (SNR), and potentially enable relaxation time estimation in a region with only a small number of voxels or even voxel wise mapping in future studies. This would be particularly beneficial for estimating the relaxation times of, e.g., tumor tissues that are often confined to small regions of interest.

## 5 Conclusion

In this study, we developed a novel 3D CRT based relaxation time MRSI sequence, which allows to measure tissue‐specific relaxation times with sub‐millimeter isotropic resolution. Using this sequence, relaxation time constants of ^2^H‐labeled resonances (Glc and Glx) following oral administration of ^2^H‐labeled Glc were determined for the first time in GM and WM matter dominated regions separately at 7 T. The versatile sequence is not limited to a specific nucleus and could help to detect local variations in relaxation times, as often observed for certain pathologies and ultimately improving accuracy of concentration estimation in future studies.

## Consent

Written informed consent was obtained from all participants.

## Conflicts of Interest

R. Lanzenberger received investigator‐initiated research funding from Siemens Healthcare regarding clinical research using PET/MR. He is a shareholder of the start‐up company BM Health GmbH since 2019.

## Supporting information


**Supplementary Table 1.** Minimum Reporting Standards for in vivo MR Spectroscopy.
**Supplementary Table 2.** Overview of SNR, FWHM number of averaged voxels and averaged voxel volume for GM and WM dominated regions for scans without (water) and after (Glc/Glx) oral administration of ^2^H Glc.
**Supplementary Figure 1** Schematic illustration in 2D showing the k‐space weighting of simulated data for k‐space reordered sampling (left) and conventional consecutive sampling (right) for a 35% decrease in metabolite levels. For k‐space reordered sampling, data is weighted using a spherical, linearly decreasing weighting function, where each k‐space point maintains the same weight across all *T*_I_/*T*_E_s, simulating repeated sampling of each ring trajectory with different *T*_I_/*T*_E_s. In contrast, for conventional consecutive sampling, each k‐space point is weighted with a linearly decreasing or increasing vector, simulating separate encoding of the k‐space for each *T*_I_/*T*_E_.
**Supplementary Figure 2.** Averaged time courses of Glc (red) and Glx (green) levels obtained from temporally interleaved unlocalized FID acquisitions during *T*_1_ (top) and *T*_2_ (bottom) measurements. Metabolite concentrations were normalized to the first timepoint and are illustrated as mean ± STD. Metabolite concentrations remained stable throughout the measurement period, with a slight mean decrease and increase in Glc and Glx levels, respectively.
**Supplementary Figure 3.** Exponential fits of *T*_1_ (a) and *T*_2_ (b) relaxation times of synthetic data modeled with two compartments sGM and sWM and known relaxation time values. Simulations included linearly increasing/decreasing metabolite levels (±35%) throughout the measurement, with k‐space encoding in two ways: the k‐space reordered approach (middle column), representing encoding of our proposed sequence, and conventional approach (right column), simulating separate acquisition of k‐space for *T*_I_/*T*_E_. Relaxation times obtained from each method were compared with the gold standard with stable metabolite levels (GS, left column). Similar relaxation times with a minimal difference of 0–3% between the GS and our k‐space reordered approach demonstrate the robustness of our sequence against fluctuating metabolite levels, while relaxation times from the conventional approach deviated from the GS (by 22–44%).

## Data Availability

Data generated by postprocessing (i.e., LCModel basis sets, script files for data plotting) are available from the corresponding author on reasonable request for research purposes only.
