# Supplementary material for: Concentric Ring Trajectory Sampling With k‐Space Reordering Enables Assessment of Tissue‐Specific T 1 and T 2 Relaxation for 2H‐Labeled Substrates in the Human Brain at 7 T
Source: NMR Biomed. 2024 Dec 19;38(2):e5311. doi: 10.1002/nbm.5311 (PMC11659634; doi:10.1002/nbm.5311)
Supplement: Supplementary file 1 — Supplementary Table 1. Minimum Reporting Standards for in vivo MR Spectroscopy. Supplementary Table 2. Overview of SNR, FWHM number of averaged voxels and averaged voxel volume for GM and WM dominated regions for scans without (water) and after (Glc/Glx) oral administration of 2H Glc. Supplementary Figure 1 Schematic illustration in 2D showing the k‐space weighting of simulated data for k‐space reordered sampling (left) and conventional consecutive sampling (right) for a 35% decrease in metabolite levels. For k‐space reordered sampling, data is weighted using a spherical, linearly decreasing weighting function, where each k‐space point maintains the same weight across all T I/T Es, simulating repeated sampling of each ring trajectory with different T I/T Es. In contrast, for conventional consecutive sampling, each k‐space point is weighted with a linearly decreasing or increasing vector, simulating separate encoding of the k‐space for each T I/T E. Supplementary Figure 2. Averaged time courses of Glc (red) and Glx (green) levels obtained from temporally interleaved unlocalized FID acquisitions during T 1 (top) and T 2 (bottom) measurements. Metabolite concentrations were normalized to the first timepoint and are illustrated as mean ± STD. Metabolite concentrations remained stable throughout the measurement period, with a slight mean decrease and increase in Glc and Glx levels, respectively. Supplementary Figure 3. Exponential fits of T 1 (a) and T 2 (b) relaxation times of synthetic data modeled with two compartments sGM and sWM and known relaxation time values. Simulations included linearly increasing/decreasing metabolite levels (±35%) throughout the measurement, with k‐space encoding in two ways: the k‐space reordered approach (middle column), representing encoding of our proposed sequence, and conventional approach (right column), simulating separate acquisition of k‐space for T I/T E. Relaxation times obtained from each method were compared with the gold standa [file NBM-38-e5311-s001.docx]

# Supplementary material

| *Minimum Reporting Standards in MR Spectroscopy checklist (according to Lin et al. NMR Biomed 2021)* | |
| --- | --- |
| **1. Hardware** |  |
| *a. Field strength [T]* | 7T |
| *b. Manufacturer* | Siemens |
| *c. Model (software version if available)* | Magnetom dot Plus |
| *d. RF coils: nuclei (transmit/ receive), number of channels, type, body part* | ^2^H/^1^H dual tuned quadrature birdcage head coil, transmit/receive, 1 channel, (Stark Contrast MRI Coils Research, Germany) |
| *e. Additional hardware* | N/A |
| **2. Acquisition** |  |
| *a. Pulse sequence* | 3D FID-acquire density-weighted concentric ring trajectory (CRT) MRSI with interleaved Inversion Recovery / Hahn spin-echo acquisitions, unlocalized FID-acquire reference scans |
| *b. Volume of Interest (VOI) locations* | whole-brain, unlocalized excitation using rectangular RF pulse with 90° flip angle |
| *c. Nominal VOI size [cm^3^, mm^3^]* | CRT MRSI: FOV 200x200x192 mm^3^ |
| *d. Repetition Time (TR), Echo Time (TE) [ms, s]* | CRT and FID: acquisition delay : 2ms (T_1_), 0ms (T_2_)  Water:   - CRT: TR_T1/T2_ = 900/400ms; TI: 5, 50, 450, 650, 900ms; TE: 6, 10, 15, 20, 30, 40, 60ms - FID: TR = 1500ms; TI: 5, 15, 50, 100, 150, 250, 450, 650, 900, 1200ms; TE: 6, 10, 15, 20, 25, 30, 40, 45, 50, 60ms   Glc/Glx:   - CRT: TR_T1/T2_ = 500/400ms; TI: 5, 15, 70, 150, 250, 500ms; TE: 6, 15, 25, 35, 45, 65, 85, 100ms - FID: TR = 1500ms; TI: 5, 15, 70, 150, 250, 450, 650, 800, 1000, 1200ms; TE: 6, 15, 35, 45, 65, 85, 100, 120, 150, 200ms |
| *e. Total number of Excitations or acquisitions per spectrum* | CRT MRSI: 27 circles + averaged over GM or WM dominated regions |
| *In time series for kinetic studies* | N/A |
| *i.         Number of Averaged spectra (NA) per time-point* | N/A |
| *ii.       Averaging method (e.g. block-wise or moving average)* | N/A |
| *iii.      Total number of spectra (acquired / in time-series)* | N/A |
| *f. Additional sequence parameters (spectral width in Hz, number of spectral points, frequency offsets); If STEAM: Mixing Time TM; If MRSI: 2D or 3D, FOV in all directions, matrix size, acceleration factors* | CRT MRSI: BW: 380 Hz, 148/96 spectral points (T_1_/T_2_), 22x22x21  FID : BW : 500Hz, 128 spectral points |
| *g. Water Suppression Method* | No water suppression |
| *h. Shimming Method, reference peak, and thresholds for “acceptance of shim” chosen* | Standard shim + manual adjustment, ^1^H water peak < 40 Hz, ^2^H water peak < 30 Hz Region: whole brain |
| *i. Triggering or motion correction method (respiratory, peripheral, cardiac triggering, incl. device used and delays)* | - |
| **3. Data analysis methods and outputs** |  |
| *a. Analysis software* | LCModel 6.3-1 |
| *b. Processing steps deviating from quoted reference or product* | N/A |
| *c. Output measure (e.g. absolute concentration, institutional units, ratio)* | - |
| *d. Quantification references and assumptions, fitting model assumptions* | Simulated in NMRScope-B |
| **4. Data Quality** |  |
| *a. Reported variables (SNR, Linewidth (with reference peaks))* | SNR and linewidths reported see Table S2 |
| *b. Data exclusion criteria* | tissue dominance threshold 40% and GM/WM ratio > 1.5 |
| *c. Quality measures of postprocessing Model fitting (e.g. CRLB, goodness of fit, SD of residual)* | CRLB |
| *d. Sample Spectrum* | See Figure 2 |

**Supplementary Table 1:** Minimum Reporting Standards for in vivo MR Spectroscopy

|  |  | **GM** | | | |  | | **WM** | | | | | | | |  |
| --- | --- | --- | --- | --- | --- | --- | --- | --- | --- | --- | --- | --- | --- | --- | --- | --- |
|  |  | **SNR** | **FWHM [Hz]** | **# averaged voxels** | **averaged voxel volume [ml]** | |  | | **SNR** | | **FWHM [Hz]** | | **# averaged voxels** | | **averaged voxel volume [ml]** | |
| **water** | *T*_1_ | 56 ± 6 | 10 ± 3 | 992 ± 107 | 448 ± 57 |  | | 42 ± 12 | | 9 ± 6 | | 414 ± 13 | | 215 ± 9 | |  |
|  | *T*_2_ | 57 ± 15 | 12 ± 6 |  |  |  | | 44 ± 14 | | 9 ± 3 | |  |  |  |  |  |
|  |  |  |  |  |  |  | |  | |  | |  | |  | |  |
| **Glc/Glx** | *T*_1_ | 65 ± 23 | 14 ± 0 | 888 ± 77 | 391 ± 66 |  | | 63 ± 12 | | 10 ± 4 | | 404 ± 17 | | 205 ± 12 | |  |
|  | *T*_2_ | 67 ± 21 | 9 ± 2 | 910 ± 62 | 417 ± 37 |  | | 52 ± 11 | | 5 ± 2 | | 423 ± 41 | | 220 ± 19 | |  |

**Supplementary Table 2:** Overview of SNR, FWHM number of averaged voxels and averaged voxel volume for GM and WM dominated regions for scans without (water) and after (Glc/Glx) oral administration of ²H Glc.


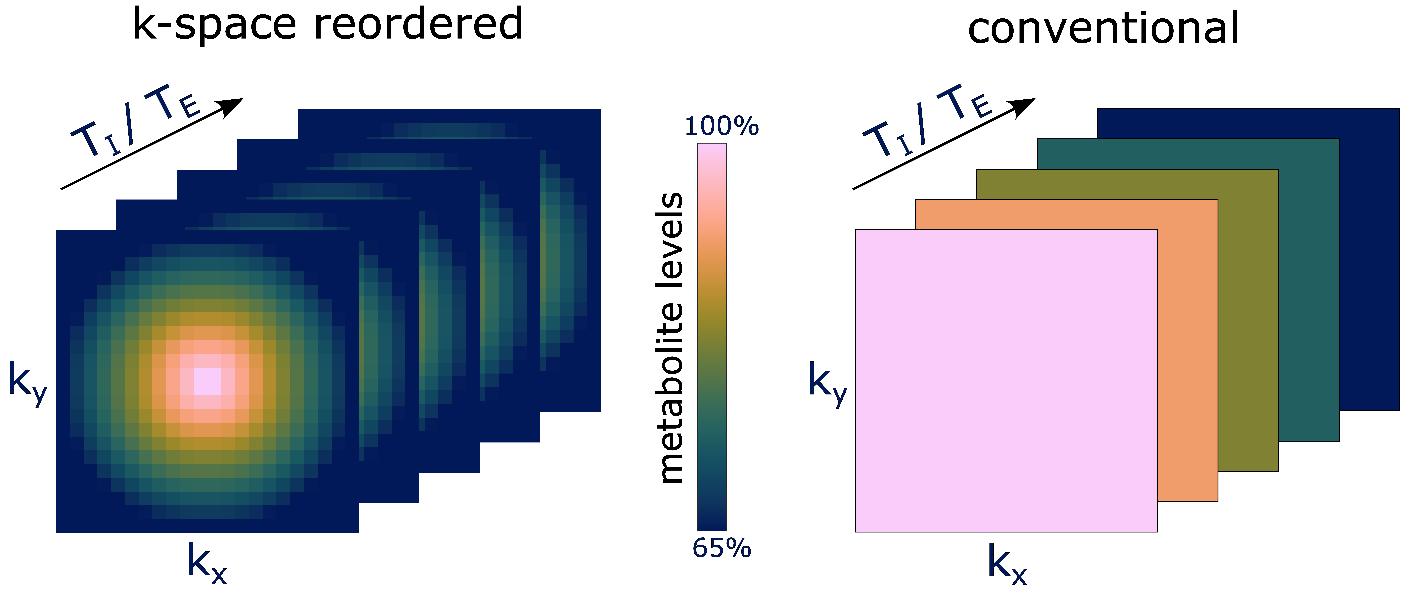


**Supplementary Figure 1:** Schematic illustration in 2D showing the k-space weighting of simulated data for k-space reordered sampling (left) and conventional consecutive sampling (right) for a 35% decrease in metabolite levels. For k-space reordered sampling, data is weighted using a spherical, linearly decreasing weighting function, where each k-space point maintains the same weight across all *T*_I_/*T*_E_s, simulating repeated sampling of each ring trajectory with different *T*_I_/*T*_E_s. In contrast, for conventional consecutive sampling, each k-space point is weighted with a linearly decreasing or increasing vector, simulating separate encoding of the k-space for each *T*_I_/*T*_E_.


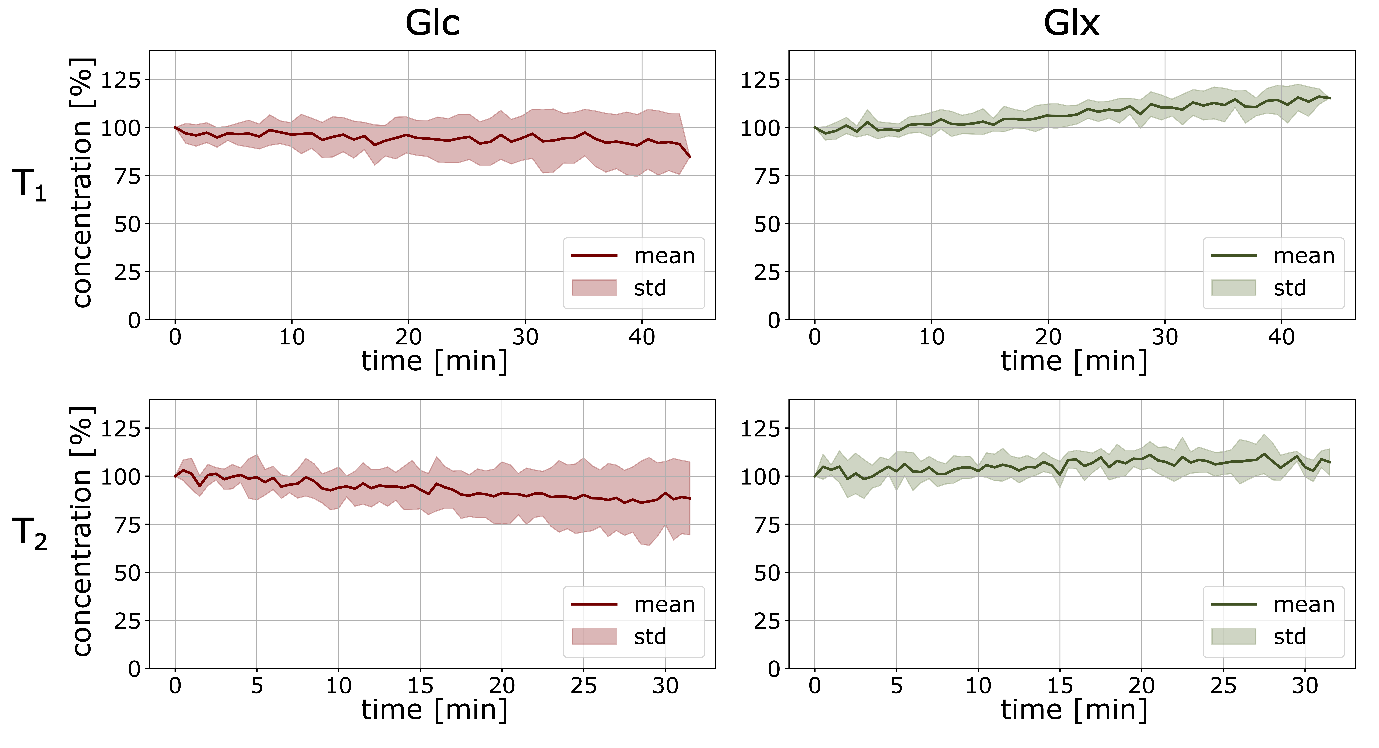


**Supplementary Figure 2:** Averaged time courses of Glc (red) and Glx (green) levels obtained from temporally interleaved unlocalized FID acquisitions during *T*_1_ (top) and *T*_2_ (bottom) measurements. Metabolite concentrations were normalized to the first timepoint and are illustrated as mean±std. Metabolite concentrations remained stable throughout the measurement period, with a slight mean decrease and increase in Glc and Glx levels, respectively.


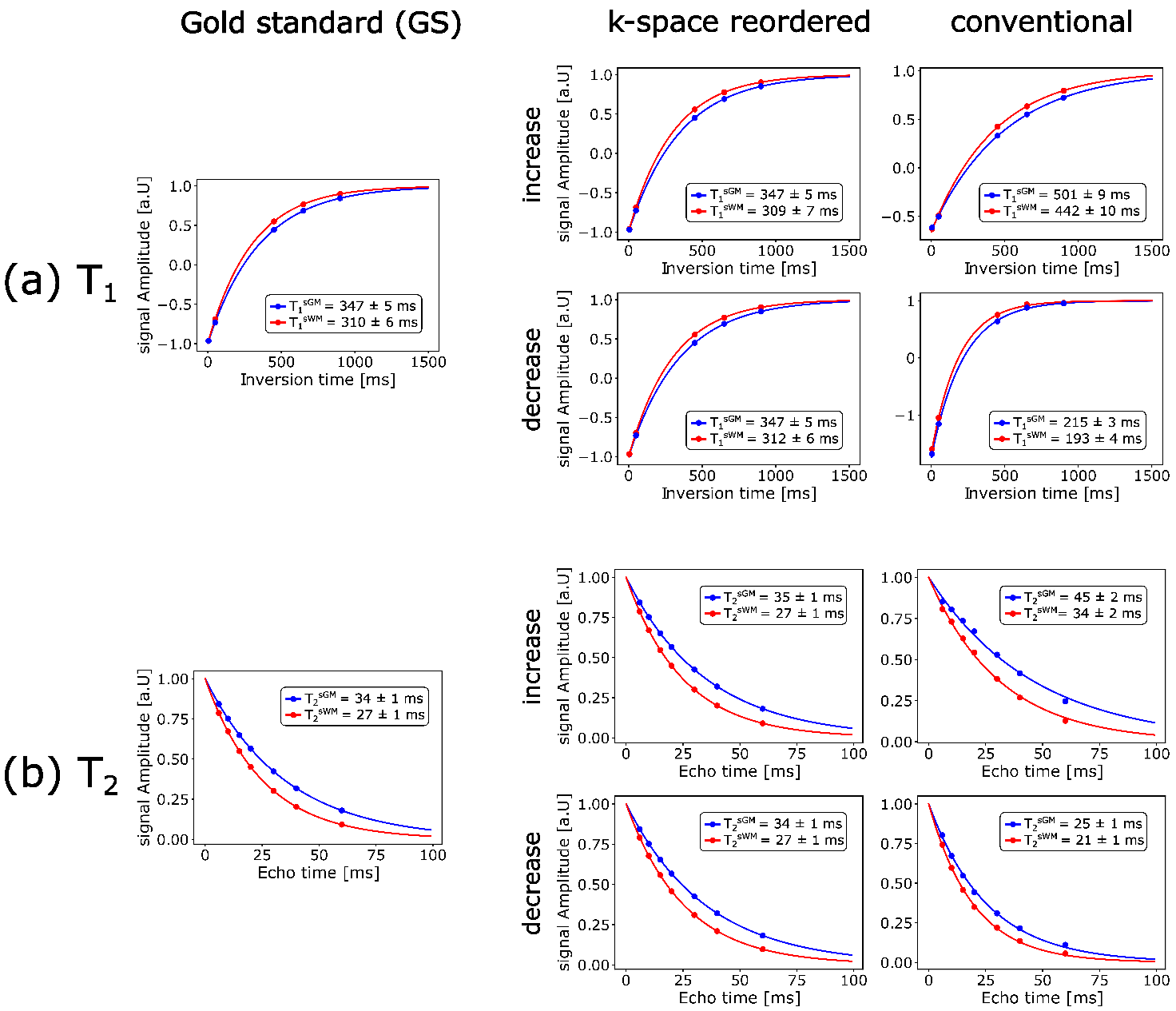


**Supplementary Figure 3:** Exponential fits of *T*_1_ (a) and *T*_2_ (b) relaxation times of synthetic data modeled with two compartments sGM and sWM and known relaxation time values. Simulations included linearly increasing/decreasing metabolite levels (±35%) throughout the measurement, with k-space encoding in two ways: the k-space reordered approach (middle column), representing encoding of our proposed sequence, and conventional approach (right column), simulating separate acquisition of k-space for *T*_I_/*T*_E_. Relaxation times obtained from each method were compared with the gold standard with stable metabolite levels (GS, left column). Similar relaxation times with a minimal difference of 0-3% between the GS and our k-space reordered approach demonstrate the robustness of our sequence against fluctuating metabolite levels, while relaxation times from the conventional approach deviated from the GS (by 22-44%).
